# Telomere biology and telomerase mutations in cirrhotic patients with hepatocellular carcinoma

**DOI:** 10.1371/journal.pone.0183287

**Published:** 2017-08-16

**Authors:** Flávia S. Donaires, Natália F. Scatena, Raquel M. Alves-Paiva, Joshua D. Podlevsky, Dhenugen Logeswaran, Barbara A. Santana, Andreza C. Teixeira, Julian J.-L. Chen, Rodrigo T. Calado, Ana L. C. Martinelli

**Affiliations:** 1 Department of Genetics, University of São Paulo at Ribeirão Preto Medical School, Ribeirão Preto, São Paulo, Brazil; 2 Department of Internal Medicine, Divisions of Hematology and Gastroenterology, University of São Paulo at Ribeirão Preto Medical School, Ribeirão Preto, São Paulo, Brazil; 3 School of Molecular Sciences, Arizona State University, Tempe, Arizona, United States of America; Tulane University Health Sciences Center, UNITED STATES

## Abstract

Telomeres are repetitive DNA sequences at linear chromosome termini, protecting chromosomes against end-to-end fusion and damage, providing chromosomal stability. Telomeres shorten with mitotic cellular division, but are maintained in cells with high proliferative capacity by telomerase. Loss-of-function mutations in telomere-maintenance genes are genetic risk factors for cirrhosis development in humans and murine models. Telomerase deficiency provokes accelerated telomere shortening and dysfunction, facilitating genomic instability and oncogenesis. Here we examined whether telomerase mutations and telomere shortening were associated with hepatocellular carcinoma (HCC) secondary to cirrhosis. Telomere length of peripheral blood leukocytes was measured by Southern blot and qPCR in 120 patients with HCC associated with cirrhosis and 261 healthy subjects. HCC patients were screened for telomerase gene variants (in *TERT* and *TERC*) by Sanger sequencing. Age-adjusted telomere length was comparable between HCC patients and healthy subjects by both Southern blot and qPCR. Four non-synonymous *TERT* heterozygous variants were identified in four unrelated patients, resulting in a significantly higher mutation carrier frequency (3.3%) in patients as compared to controls (*p* = 0.02). Three of the four variants (T726M, A1062T, and V1090M) were previously observed in patients with other telomere diseases (severe aplastic anemia, acute myeloid leukemia, and cirrhosis). A novel *TERT* variant, A243V, was identified in a 65-year-old male with advanced HCC and cirrhosis secondary to chronic hepatitis C virus (HCV) and alcohol ingestion, but direct assay measurements *in vitro* did not detect modulation of telomerase enzymatic activity or processivity. In summary, constitutional variants resulting in amino acid changes in the telomerase reverse transcriptase were found in a small proportion of patients with cirrhosis-associated HCC.

## Introduction

Telomeres cap and protect the ends of linear chromosomes from aberrant double-stranded DNA repair and detrimental end-to-end fusions, as well as ensuring proper genetic partitioning into daughter cells. The structure of telomeres is composed of hexanucleotide repeats bound specifically by ‘shelterin’ proteins [1]. Telomeres function as ‘mitotic clocks’ and shorten with subsequent mitotic cell divisions. Upon reaching a critically short length, a cellular signaling cascade involving p53 and p21 arrests cellular replication and induces senescence [2,3]. The telomerase enzyme complex counterbalances telomere attrition by *de novo* synthesizing telomere repeats, maintaining telomere length in high proliferative cells.

Telomerase is a ribonucleoprotein and is minimally composed of the catalytic telomerase reverse transcriptase (TERT) protein and intrinsic RNA component (TERC), which provides the template for telomere repeat synthesis. The human telomerase holoenzyme is composed of several accessory proteins, including the dyskerin protein complex [4–6]. Deleterious mutations within telomerase- and telomere-associated genes that impair telomere maintenance result in ‘telomeropathies’, a spectrum of progressive genetic diseases molecularly caused by telomere dysfunction and exemplified by dyskeratosis congenita [3,7]. Telomeropathies are degenerative diseases characterized by premature stem cell senescence, which impart an increased risk of organ failure for hematopoietic, pulmonary, mucosal, dermal, and hepatic compartments as well as a predisposition towards the development of cancer. Consistently, loss-of-function mutations in *TERT* and *TERC* genes are related to a spectrum of familial hepatic disorders [8]. Liver dysfunction in telomeropathy patients is highly variable [9–11].

Mutations within the *TERT* gene have been found to either directly damage the activity of the telomerase enzyme, or indirectly decrease the stability of the ribonucleoprotein complex [12]. Our previous analysis of the prevalence of telomerase mutations in patients with cirrhosis of diverse etiologic backgrounds—mainly alcohol-, hepatitis B- or C-induced—revealed an enrichment for *TERT* gene mutations compared to healthy controls [13]. This indicates that telomerase dysfunction may predispose to cirrhosis development in response to chronic liver damage. The persistent and increased cellular turnover from cirrhosis combined with decreased telomerase function may result in progressive telomere shortening [14] with the associated chromosomal instability that facilitates the development of cancers, such as hepatocellular carcinoma (HCC) [9].

The presence of deleterious telomerase- and telomere-associated gene mutations may serve as risk factors for cancer development in patients with chronic diseases. In the present work, we investigated the contribution of telomere dysfunction and genetic mutations in telomerase components to HCC pathogenesis in cirrhotic patients.

## Material and methods

### Patient sample collection

Blood samples were collected from a cohort of 261 healthy donors (144/117 male/female) and 120 patients with HCC secondary to hepatic cirrhosis (102/18 male/female) from the Clinical Hospital, University of São Paulo at Ribeirão Preto Medical School. Patient blood samples were collected prior to HCC treatment by chemotherapy and/or radiation. Healthy individuals were recruited from blood donors, as well as healthy child tonsillectomy patients, and healthy local volunteer study participants. All healthy controls had normal blood cell counts. Healthy control age ranged from newborn via umbilical cord to 88 years old and HCC patient age ranged from 23 to 85 years old. This study was approved by the Local Ethics Committee (Comitê de Ética em Pesquisa do Hospital das Clínicas de Ribeirão Preto– 12050/2011) with written consent from all participants or their legal guardians (16480/2012).

### DNA extraction

Genomic DNA was isolated from white blood cells using the Gentra Puregene Blood kit (Qiagen, Maryland, USA), following the manufacturer’s instructions. DNA sample integrity was analyzed by agarose gel electrophoresis prior to telomere length measurements and gene sequencing.

### Telomere length analysis

For terminal restriction fragment (TRF), the analysis was performed as previously described [15] with the TeloTAGGG Telomere Length Assay (Roche Applied Science, Mannheim, Germany). Briefly, 800 ng genomic DNA was digested by FastDigest *Hinf*I and *Rsa*I (Thermo Scientific, Waltham, MA, USA) at 37°C for 2 h. Following digestion, DNA fragments were electrophoresed for 5 h on a 0.8% agarose gel, denatured, neutralized, and transferred to a nylon membrane for Southern blot analysis with proprietary digoxigenin (DIG)-labeled probes and chemiluminescent substrates. We determined the mean TRF length based on the equation Σ(ODi)/Σ(ODi/Li), where ODi represents the chemiluminescent signal and Li corresponds to the fragment length at a given position. For each experiment, a reference sample was included.

Relative telomere length was measured by qPCR as previously described [15]. The qPCR was performed in triplicate with each 24 μL reaction comprising of 1.6 ng genomic DNA, 1× Rotor-Gene SYBR Green (manufacturer), 1× PCR Master Mix (Qiagen, Hilden, Germany), and primers: for telomere amplification, 300 nM forward (5’-CGGTTTGTTTGGGTTTGGGTTTGGGTTTGGGTTTGGGTT-3’) and reverse (5’-GGCTTGCCTTACCCTTACCCTTACCCTTACCCTTACCCT-3’), or for the single gene 36B4 300 nM forward (5’-CAGCAAGTGGGAAGGTGTAATCC-3’) and 500 nM reverse (5’ CCCATTCTATCATCAACGGGTACAA 3’). All qPCR reactions were prepared on a QIAgility automated pipettor (Qiagen, California, USA), and amplified in a Rotor-Gene Q (Qiagen) real-time PCR cycler as follows: 5 min at 95°C, and either 25 cycles of 7 s at 98°C and 10 s at 60°C (telomere), or 35 cycles of 7 s at 98°C and 10 s at 58°C (single gene). To determine the telomere length for each sample, we used the telomere to single copy gene (T/S) ratio based on the ΔCt (Ct_telomere_/Ct_single gene_). The T/S ratio for a given sample (x) was normalized to the mean T/S ratio of a reference sample (2^–(ΔCtx−ΔCtr)^ = 2^−ΔΔCt^). Each qPCR run included samples for the standard curve and two reference reactions. All sample data included had reference samples with less than 95% variation. Linear regressions to correlate telomere length measurements by TRF and qPCR were performed using R software (v3.0.3) and Prism v5 (GraphPad Software Inc, CA, USA).

### Mutation screening

*TERT* and *TERC* genes were screened for genetic variants as previously described [16]. Non-synonymous mutations (missense, nonsense, and frameshift) and mutations with an allele frequency <1% were included in this work. PCR conditions are listed in S1 Table. *In silico* prediction of the possible impact of non-synonymous variants on the structure and function of the human TERT enzyme was performed by PolyPhen-2 [17] and SIFT [18] tools. The variants were annotated using the Combined Annotation Dependent Depletion (CADD, http://cadd.gs.washington.edu/home). From this annotation, we provided the ‘CADD score’ of each variant, which corresponds to the PHRED-like scaled C score [19], a predictor of deleteriousness. We considered variants with CADD scores higher than 10 as potentially pathogenic.

### Telomerase repeat amplification protocol

Wild-type and variant telomerase were reconstituted *in vivo* in telomerase-negative WI 38 VA13 ALT-positive cells (ATCC) by transient transfection with 2 μg puc57-TERC, 2 μg pcDNA3-Flag-hTERT and Superfect Transfection Reagent (Qiagen), following the manufacturer’s instructions. Cell lysates were prepared with CHAPS buffer (TRAPeze XL Telomerase Detection Kit, Millipore), clarified by centrifugation, and protein concentration determined by Pierce BCA Protein Assay (Thermo Scientific). Reconstituted telomerase in cell lysate containing 300 ng of total protein was analyzed by the PCR-based Telomeric Repeat Amplification Protocol (TRAP) assay with the TRAPeze XL Telomerase Detection kit (Millipore), following the manufacturer’s instructions. TRAP reactions were electrophoresed on non-denaturing 10% polyacrylamide gel and stained with ethidium bromide. Telomerase activity was determined by measuring the total intensity of products on the gel using an ImageQuant imaging system and software (GE Healthcare Life Sciences). Quantification was based on TRAP assays using cell lysates from two independent transfections.

### Telomerase activity and processivity assay

Wild-type and variant telomerase were reconstituted *in vivo* in telomerase-positive HEK293FT cells (Invitrogen, Carlsbad, CA) by transient transfection with pcDNA-Flag-hTERT and pBS-U1-hTR [20]. The catalytically inactive negative control D868N mutant was appended with a 3×Flag tag in place of a single Flag in the pcDNA-Flag-hTERT vector. The reconstituted telomerase was then immuno-purified from cell lysates and analyzed by the direct primer-extension assay at physiological nucleotide concentrations, as previously validated and described [20–22]. The 10 μL direct primer-extension reaction contained 5 μM dTTP, 5 μM dATP, 5 μM dGTP, 0.165 μM α-^32^P-dGTP (3000 Ci/mmol, 10 mCi/ml, Perkin-Elmer), and 1 μM (TTAGGG)_3_ DNA primer in 1× reaction buffer (0.5 mM MgCl_2_, 50 mM Tris-HCl pH 8.3, 2 mM DTT, and 1 mM spermidine). In transfected HEK293FT cells, wild-type and variant telomerase expression was confirmed by Western blot for the Flag-tagged TERT protein with GAPDH as an internal control [23]. Telomerase activity was determined by measuring the total intensity of telomerase-generated products on the gel and normalized against the TERC level determined by Northern blot for TERC extracted from immuno-purified telomerase. Telomerase processivity was determined by the equation—ln2/(2.303×*k*), with *k* as the slope of the log plot of the intensity of the telomerase-generated major bands divided by the number of radioactive dGTP incorporations [24]. Quantification was based on four direct activity assays using cell lysates from two independent transfections.

## Results

**Fig 1:**
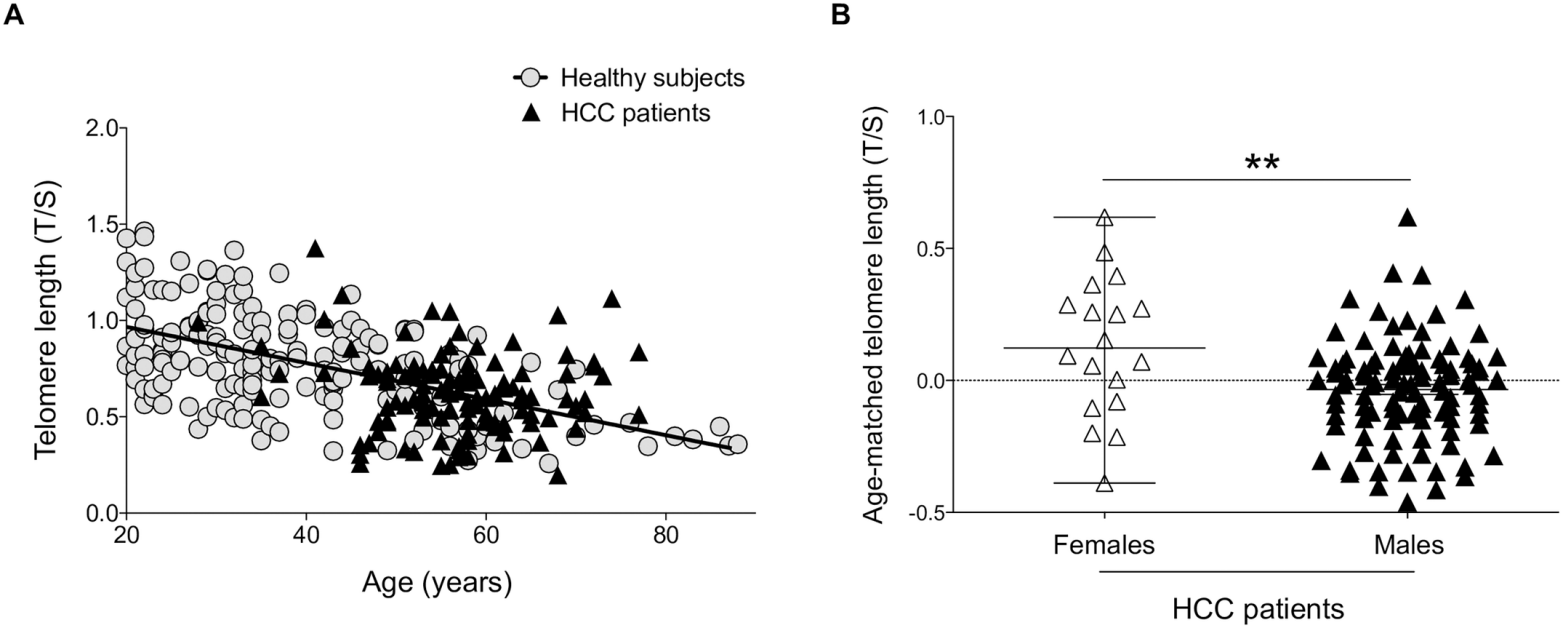
Distribution of age-matched telomere length in healthy subjects and HCC patients by qPCR. **(A)** Telomere length analysis revealed no statistical differences between HCC patients and healthy subjects analyzed by qPCR. Patients and controls were age-matched: Slope: −0.007882±0.0007139; y-intercept when x = 0: 1.080±0.02516). **(B)** Telomere length was longer in females than in males (**p = 0.01).

We assessed the telomere lengths of peripheral blood leukocytes of patients with HCC and cirrhosis. Telomere length was measured by qPCR in 261 healthy subjects with no history of liver disease or malignant formation and in 120 patients with HCC associated with cirrhosis (Fig 1A; Table 1). Cirrhosis was mainly caused by hepatitis B- or C-virus alone (11% and 29%, respectively), alcohol (17.5%), or a combination of virus infection and alcohol (32.5%). This study classified 60 patients (50%) as Child-Pugh class A, 38 (32%) as Child-Pugh class B, and 18 (15%) as Child-Pugh Class C.

**Table 1:** Demographic and clinical characteristics of cirrhotic patients with hepatocellular carcinoma.

	Frequency	Percentage	p value
Telomere length classification*			0.01
<p25%	29	24	
p25 –p50	31	26	
p50 –p75	30	25	
>p75%	30	25	
**Age (years)**			0.1
Female (39–79)			
Male (28–85)			
Median (<p50%: 59; >p50%: 58.5)			
**Gender**			<0.01
Female	18	15	
Male	102	85	
**Primary disease**			
NASH	1	1	
Cirrhosis	116	97	
Hepatitis	3	2	
**Etiology**			>0.05
Hepatitis B virus	13	11	
Hepatitis C virus	35	29	
Alcoholism	21	17.5	
Virus (B or C) + Alcoholism	39	32.5	
Cryptogenic	7	6	
NASH	4	3	
HH	1	1	
**CHILD**			0.6
A	60	50	
B	38	32	
C	18	15	
ND	4	3	
**MELD**			0.4
<10	28	23	
10–19	80	67	
20–29	6	5	
ND	6	5	
**Tumor presentation**			
Diffuse infiltrative	16	13	
Multinodular	28	23	
Uninodular	75	63	
ND	1	1	
**Milan criteria**			
No	54	45	
Yes	66	55	
**Metastasis**			
Adrenal	1	1	
Lung	2	2	
Bone	5	4	
No research	8	7	
No	104	86	
**BCLC**			0.8
A1	7	6	
A2	12	10	
A3	14	12	
A4	19	16	
B	28	23	
C	22	18	
D	18	15	

* T/S ratio; p = percentile

NASH: non-alcoholic steatohepatites; HH: Hereditary hemochromatosis; CHILD: Child-Pugh score; MELD: Model for End-Stage Liver Disease; BCLC: Barcelona-Clinic Liver Cancer; ND: follow up lost.

Telomere length was not statistically different between HCC patients and healthy subjects (*p* = 0.6), with a similar loss of telomere length with age in both groups (*p* = 0.0001) (Fig 1A). In order to validate qPCR results, we used Southern blot, the gold standard technique for telomere length, which was performed on 204 healthy subjects and 48 HCC patients, all randomly selected. Again, no significant difference in telomere length was detected between patients and healthy subjects (*p* = 0.9), and both HCC patients and healthy subjects showed significant telomere shortening according to age (*p* = 0.0001), with a mean loss of 73 and 48 base pairs (bp) per year, respectively, but there was no significant difference between the two groups (*p* = 0.2) (S1A Fig). Eighteen out of the 120 (15%) HCC patients tested were female and 102 (85%) were male. Median of telomere length was higher in females than in males when analyzed by qPCR (*p* = 0.01) (Fig 1B) and a concordant tendency for increased telomere length in women was observed by Southern blot (*p* = 0.07) (S1B Fig).

**Fig 2:**
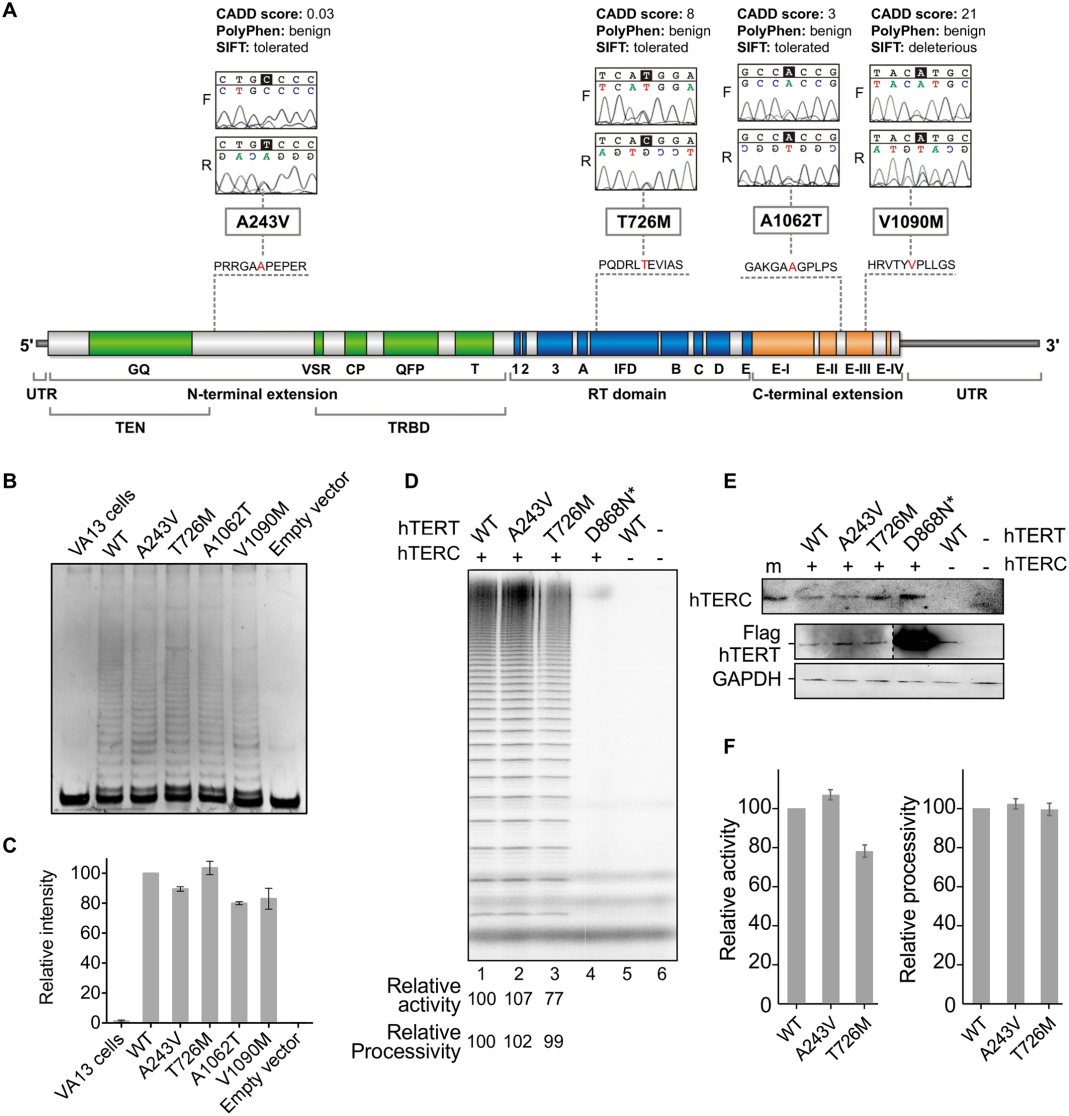
The functional consequences of variants on enzyme activity. **(A)** Four non-synonymous *TERT* heterozygous mutations (A243V, T726M, A1062T, and V1090M) were detected in HCC patients in both forward and reverse sequences; *in silico* analysis was performed by CADD, PolyPhen, and SIFT to predict the impact of each variant on the structure and function of the enzyme. The telomerase enzyme is represented in its three domains: N-terminal (green), Reverse Transcriptase (blue), and C-terminal (orange) with all described mutations in red (adapted from http://telomerase.asu.edu/diseases.html#tert). **(B)** Analysis of TERT variants’ impact on telomerase activity using PCR-based TRAP assay: representative gel image of telomeric DNA repeats generated from wild-type (WT) and variant telomerases reconstituted *in vivo*. The cell lysates for TRAP assay were obtained from reconstitution of the WT, empty, or mutated TERT expression vectors in the telomerase-negative VA13 cell line cotransfected with TERC-containing vector. No telomeric DNA repeats were obtained from lysates of VA13 cells and cells transfected with the empty vector (negative controls). **(C)** Mean intensity (and standard error) of telomeric DNA repeats quantitated from the TRAP gels. Intensities are shown relative to the WT (set as 100%). Cell lysates were obtained from two independent transfections. The TRAP assay was performed for each transfection. **(D)** Analysis of TERT variants’ impact on telomerase activity and processivity using direct assay: gel image of telomeric DNA repeats generated from WT and variant telomerases reconstituted *in vivo* and immuno-purified. The decreased total intensity of the DNA repeat products generated by variant telomerases relative to wild-type enzyme reflects slightly impaired enzymatic activity of TERT T726M. Processivity remained similar to WT for the two variants tested (A243V and T726M). The TERT mutation D868N is a negative control, catalytically defective in one of the three essential aspartic acid residues for reverse transcription. **(E)** Northern blot for TERC levels from immuno-purified telomerases and Western blot for TERT expression levels in cells. Western blot performed with anti-Flag and anti-GAPDH antibodies for ectopically expressed Flag-tagged TERT and endogenous GAPDH, respectively. The greater intensity of the catalytically inactive D868N mutant was due to the presence of a 3×Flag tag in place of a single Flag present for the WT and variant TERT proteins. **(F)** Mean telomerase activity and processivity derived from four independent activity assays. Enzymes were purified from cell lysates from two separate transfections.

Sequencing of the *TERT* gene from 120 HCC patients revealed four non-synonymous *TERT* heterozygous variants: A243V, T726M, A1062T, and V1090M from four unrelated patients (Fig 2A, Table 2), demonstrating a significantly higher mutation carrier frequency in HCC patients (3.3%) as compared to 198 healthy subjects (*p* = 0.02, by Fisher’s exact test) previously screened for *TERT* gene variants in Calado *et al*. [25]. Synonymous and intronic variants identified in our cohort are provided in S2 Table. No *TERC* variants were detected in HCC patients.

**Table 2:** Clinical data and etiology of cirrhosis in four HCC patients harboring *TERT* mutations.

ID	Age (yrs)	Stage	Exon	Mutation	Etiology	Follow up
#1 HCC	65	Okuda III, BCLC D	2	A243V	HCV+EtOH	Tx; death not related to HCC
#2 HCC	66	Okuda I, BCLC A1	6	T726M	EtOH	Tx; No recurrent HCC
#3 HCC	47	Okuda III, BCLC D	15	A1062T	HBV	None
#4 HCC	56	Okuda I, BCLC A1	15	V1090M	HBV	Tx; death not related to HCC

ID: patient identification; HCC: hepatocellular carcinoma; BCLC: Barcelona-Clinic Liver Cancer; HBV: hepatitis B virus; HCV: hepatitis C virus; EtOH: alcoholism; Tx: liver transplant; None: follow up discontinued.

For the control sample size in our previous study (n = 528), the statistical power to identify a single-nucleotide polymorphism (SNP) with a minor allele frequency of 1% would be approximately 90% [13]. Specifically for the *TERT* A1062T variant, we previously screened 2,000 healthy controls and found an allele frequency of 0.007 [13]. Herein aggregate data were accessed from the Exome Aggregation Consortium (ExAC) of more than 100,000 sequenced alleles, and 190 missense variants in *TERT* were found, significantly below the 469 missense variants expected, indicating that the gene is highly conserved in humans.

Three of the four missense *TERT* gene mutations, T726M, A1062T, and V1090M, within our sample cohort were previously described by Liang *et al*. [26], Calado *et al*. [13], Calado *et al*. [25], and Yamaguchi *et al*. [16], in patients with other telomere diseases: severe aplastic anemia, acute myeloid leukemia, and cirrhosis. T726M was observed in 6 out of 121,390 alleles, of which one was from African and one was from European (non-Finnish) populations (minor allele frequency of 5×10^−5^) in ExAC. This variant has been previously described in one patient with aplastic anemia and in her asymptomatic father [26], as well as in 2 out of 209 smokers with emphysema [22]. A1062T was found in 1,496 out of 113,542 alleles with 13 homozygotes (minor allele frequency of 1×10^−2^), mostly from European populations. In the Latin American population, this variant was found in 33 out of 10,973 alleles (minor allele frequency of 3×10^−3^) in ExAC. A1062T has been previously described in healthy individuals, but its frequency increases in patients with aplastic anemia [16], acute myeloid leukemia [25], and cirrhosis [13]. V1090M was observed in 7 out of 119,373 alleles (3 in African, 3 in Latin American and 1 in European populations; allele frequency of 6×10^−5^) in ExAC. This variant has been previously described as pathogenic in patients with severe aplastic anemia [16]. We found this variant just as deleterious by SIFT, and was predicted to be among the 1% most deleterious substitutions by CADD (score = 21); PolyPhen has categorized this variant as ‘benign’ (Fig 2A), which indicates that *in silico* predictions are useful, but insufficient to recapitulate the functional impact *in vivo*.

The A243V variant described here has not been previously reported in ExAC. This novel heterozygous *TERT* variant was identified in a 65-year-old male with advanced HCC and cirrhosis secondary to chronic hepatitis C infection and alcohol ingestion (Table 2). *In silico* prediction of possible impact of this amino acid substitution on the structure and function of the human TERT enzyme was performed, demonstrating a putative benign impact on telomerase activity (CADD score = 0.03; Fig 2A). To confirm the *in silico* prediction, two *in vitro* assays were performed: PCR-based TRAP and direct assay (Fig 2B–2F). The relative intensity of telomeric DNA repeats quantitated from the TRAP gel showed little to no modulation in telomerase activity for this variant, as well as for the other three variants, compared to the wild-type telomerase (Fig 2B and 2C). A direct primer extension assay was performed for the *TERT* A243V and T726M variants (Fig 2D). The catalytically inactive D868N mutant, which lacks one of the three essential aspartic acids, was included as control, along with the wild-type *TERT* without *TERC*. These mutant telomerases were immuno-purified from HEK293FT cell lysates for activity and processivity analysis. The *TERT* T726M mutant displayed decreased activity, consistent with previous reports [22,25]. Conversely, the direct assay showed a minor increase in activity and processivity of the novel A243V variant compared to the wild-type enzyme, suggesting that the amino acid substitution had little impact on TERT enzymatic function (Fig 2D and 2F). The A243V variant did not affect TERT protein expression or telomerase ribonucleoprotein formation, evidenced by similar levels of TERT in cell lysates and co-immunoprecipitated TERC (Fig 2E).

**Fig 3:**
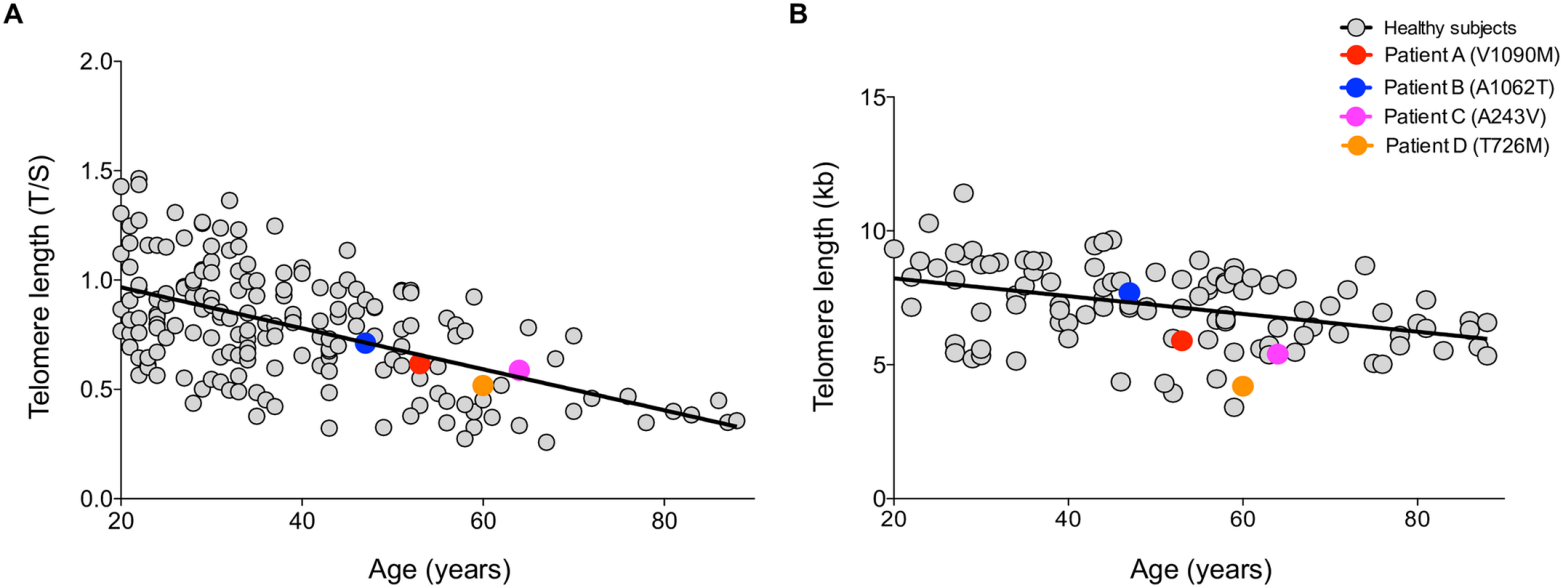
Distribution of age-matched telomere length in healthy subjects and HCC mutated patients. Healthy subjects (n = 261) are represented as grey circles; patients carrying *TERT* variants (n = 4) are represented as colored circles. Telomere length given as **(A)** T/S ratio by qPCR **(B)** and kilobases (kb) by Southern blot.

Telomere length was performed on the patients carrying the *TERT* variants and no significant difference between patients and healthy controls was observed either by qPCR (*p* = 0.7) or Southern blot (*p* = 0.1) (Fig 3A and 3B, respectively). Linear regression was used to estimate the correlation between qPCR and Southern blot, resulting in r^2^ = 0.6, r^2^ = 0.4, and r^2^ = 0.9 in controls (n = 76), HCC patients (n = 51), and *TERT*-mutant HCC patients (n = 4), respectively (S2 Fig). Loss of 48 bp/year by TRF was found in control group. Both techniques showed high proximity values, ensuring the reliability of telomere length measurement.

## Discussion

In the present study, we found that constitutional *TERT* missense variants were found in 3.3% of patients diagnosed with HCC associated with cirrhosis, but peripheral blood leukocyte telomere length did not correlate with HCC development. A previous study reported on one case of HCC in a patient with cirrhosis associated with non-alcoholic fatty liver carrying a *TERT* mutation [27].

Three out of the four variants that we have found in our HCC cohort were previously associated with telomeropathies in other studies [13,16,25,26]. The T726M *TERT* variant was observed in a Japanese patient with very severe aplastic anemia. The patient, a 9-year-old girl, presented very short telomeres whereas her father, also carrying the mutation, was asymptomatic and presented normal telomere length, suggesting disease anticipation [26]. The A1062T variant was described by Yamaguchi *et al*. [16]. Subsequently, Calado *et al*. [25] described A1062T with an allele frequency 3-fold enriched in patients with acute myeloid leukemia. The A1062T variant was also found in higher frequency in cirrhotic patients [13]. The V1090M variant, located in the C-terminal of TERT, was described in a 64-year-old Hispanic patient with severe aplastic anemia and short telomeres (allele frequency of 0.005) [16]. The PCR-based TRAP assay was previously performed for these three *TERT* variants (revised by Zaug *et al*. [28]). Xin *et al*. [29] reconstituted the T726M variant *in vivo* and reported no significant modulation in telomerase activity, in agreement with our findings. The A1062T variant was reconstituted in VA13 cells in a previous study [25] and the telomerase activity was 60% compared to the wild-type. Here we found reduced telomeric DNA repeat intensity in TRAP for this variant when compared to wild-type (80%). Yamaguchi *et al*. showed that in patient’s peripheral-blood mononuclear cells the V1090M variant displayed approximately 50% activity when compared with unrelated controls. *In vitro* this variant exhibited less than 1% activity [16]. In contrast, we have detected a slightly reduced activity of this variant in our TRAP assay (80% activity of the wild-type), although our *in silico* prediction partially agreed with previous studies.

A novel variant (A243V) was also found, located at N-terminal region of *TERT*. Mutations in this region usually modify the enzymatic processivity, correlating with shorter telomeres in cells [30,31]. However, in the present work, the patient carrying this variant had telomere lengths in the normal range and the A243V substitution did not appear to modulate telomerase ability to elongate telomeres both *in silico* and *in vitro*.

Zaug *et al*. [28] have comprehensively analyzed the effects of telomere disease-associated *TERT* variants on telomerase enzymatic activity and processivity through direct assay. The direct enzyme assay assesses the ability of telomerase to elongate telomeres without PCR amplification, as performed in the TRAP assay, avoiding potential artifacts. In their work, three of the four *TERT* variants we describe here were tested and they found that *TERT* T726M and V1090M did not significantly change processivity, whereas A1062T tended to reduce processivity. Although they did not test A243V, other variants in the same region (DAT, in the TEN domain) were tested and did not modulate processivity, but they observed dissociation between *in vivo* and *in vitro* activities of telomerase in this region. The authors postulate that whereas some mutations clearly abolish telomerase enzymatic activity, it is possible that small reductions in telomerase function are sufficient to shorten telomeres over many years. They also suggest that, alternatively, mutations may interfere with other telomerase functions, such as its recruitment to telomeres or other proteins, which are necessary *in vivo* but not easily revealed *in vitro* [28]. The *TERT* variants that were observed in our cohort may be responsible for disturbing telomerase homeostasis *in vivo*, but we were not able to detect this phenomenon in our *in vitro* assays.

In conclusion, our observations indicate that *TERT* variants are observed in a small number of patients with HCC associated with cirrhosis.

## Supporting information

S1 Fig Distribution of age-matched telomere length in healthy subjects and HCC patients by Southern blot. **(A)** Telomere length analysis revealed no statistical differences between HCC patients and healthy subjects as analyzed by Southern blot. **(B)** Telomere length by Southern blot technique showed a tendency of longer telomere length in females than in males, although no statistical difference was detected in this analysis (*p* = 0.1).
(TIF)

S2 Fig Correlation between qPCR and Southern blot techniques. Leukocyte telomere length was measured in healthy subjects and HCC patients. **(A)** Telomere length from 76 healthy subjects. Linear regression plots of qPCR (T/S ratio) × TRF analysis (kb) measurements; solid line represents the data best fit (r^2^ = 0.5); **(B)** Telomere length from 49 HCC patients. Linear regression plots of qPCR (T/S ratio) × TRF analysis (kb) measurements; solid line represents the data best fit (r^2^ = 0.4); **(C)** Telomere length from HCC mutated patients. Linear regression plots of qPCR (T/S ratio) × TRF analysis (kb) measurements; solid line represents the data best fit (r^2^ = 0.9).
(TIF)

S1 Table PCR conditions for *TERT* and *TERC* amplifications. (DOC)

S2 Table Synonymous polymorphisms in *TERT* gene of HCC patients. (DOC)
