# Supplementary figures and images for: Telomere biology and telomerase mutations in cirrhotic patients with hepatocellular carcinoma
Source: PLoS One. 2017 Aug 16;12(8):e0183287. doi: 10.1371/journal.pone.0183287 (PMC5558955; doi:10.1371/journal.pone.0183287)

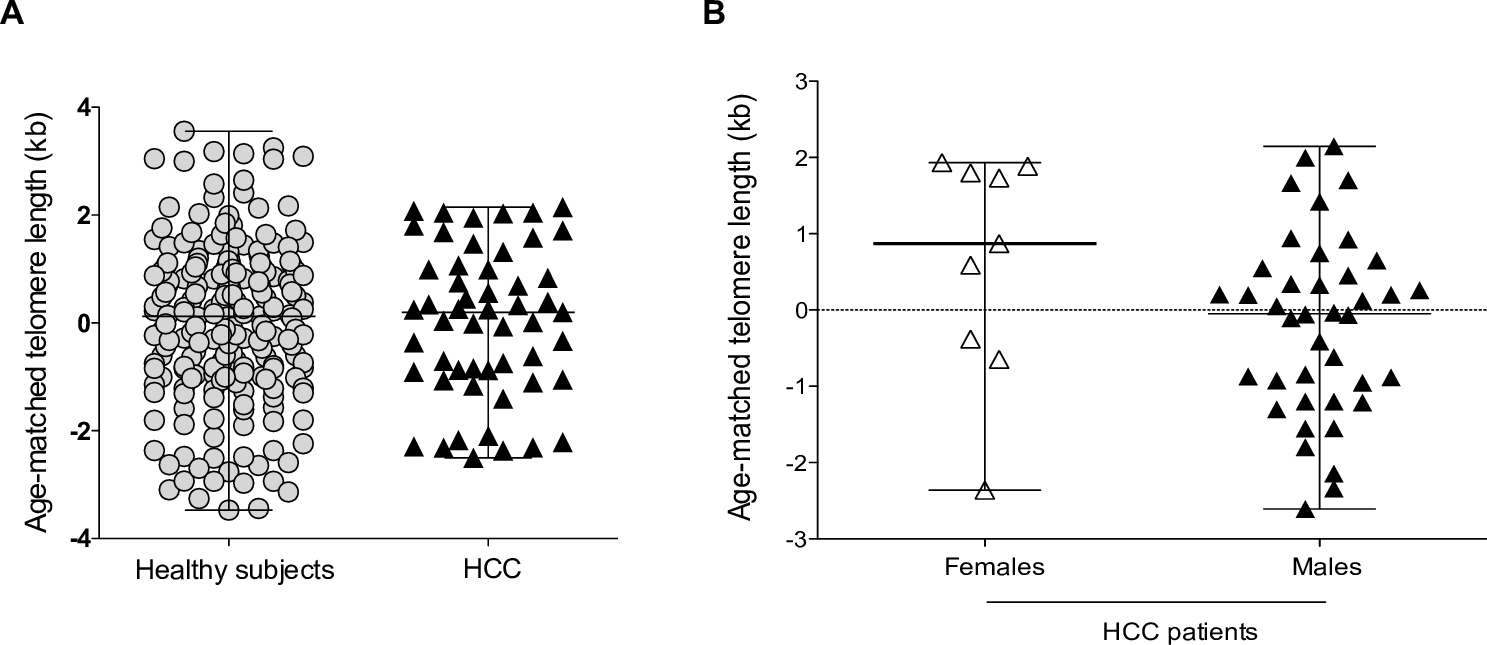

Supplement: S1 Fig — (A) Telomere length analysis revealed no statistical differences between HCC patients and healthy subjects as analyzed by Southern blot. (B) Telomere length by Southern blot technique showed a tendency of longer telomere length in females than in males, although no statistical difference was detected in this analysis (p = 0.1). (TIF) [file pone.0183287.s001.tif]

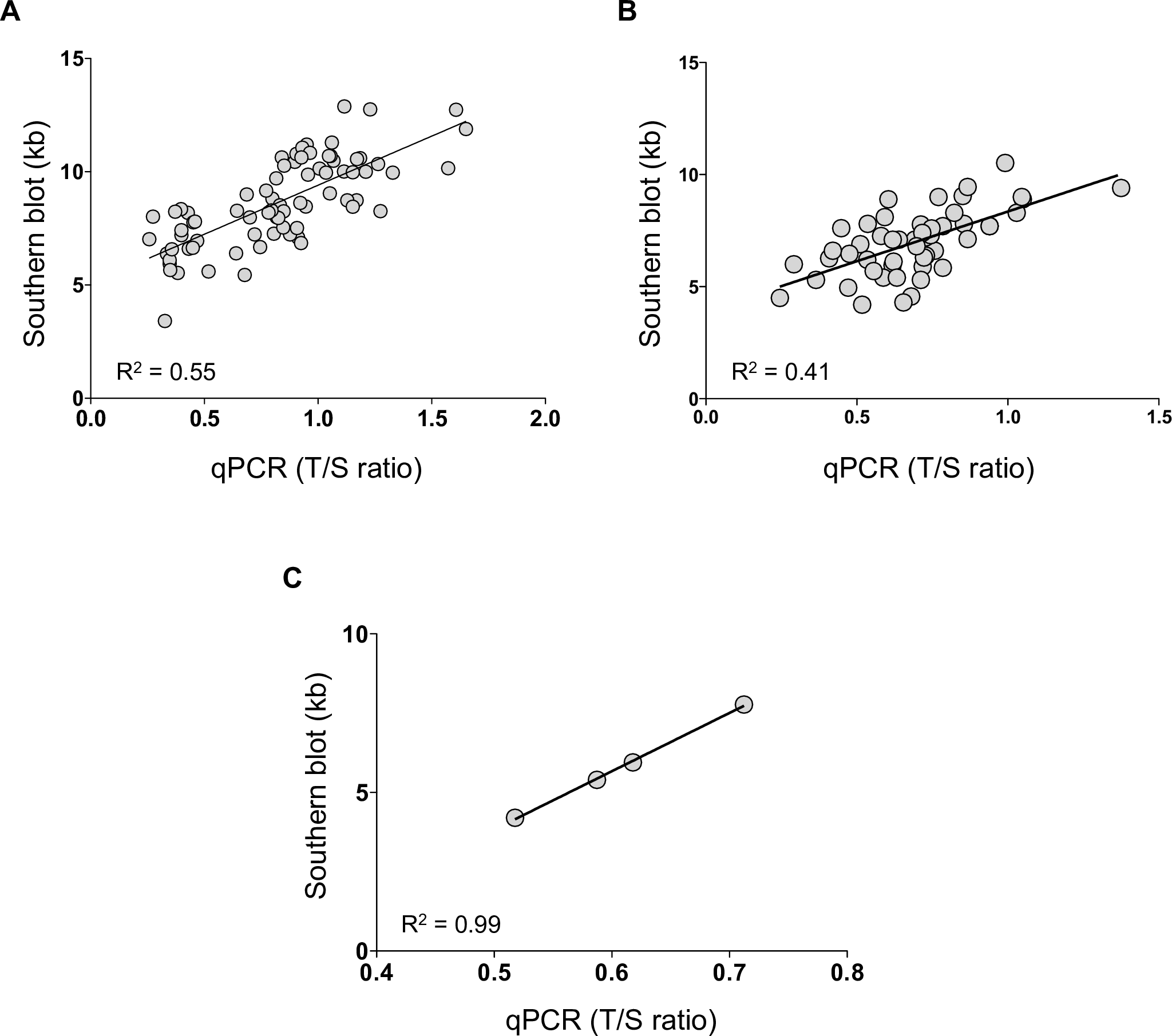

Supplement: S2 Fig — Leukocyte telomere length was measured in healthy subjects and HCC patients. (A) Telomere length from 76 healthy subjects. Linear regression plots of qPCR (T/S ratio) × TRF analysis (kb) measurements; solid line represents the data best fit (r2 = 0.5); (B) Telomere length from 49 HCC patients. Linear regression plots of qPCR (T/S ratio) × TRF analysis (kb) measurements; solid line represents the data best fit (r2 = 0.4); (C) Telomere length from HCC mutated patients. Linear regression plots of qPCR (T/S ratio) × TRF analysis (kb) measurements; solid line represents the data best fit (r2 = 0.9). (TIF) [file pone.0183287.s002.tif]
