# Supplementary material for: Telomere biology and telomerase mutations in cirrhotic patients with hepatocellular carcinoma
Source: PLoS One. 2017 Aug 16;12(8):e0183287. doi: 10.1371/journal.pone.0183287 (PMC5558955; doi:10.1371/journal.pone.0183287)
Supplement: S1 Table — (DOC) [file pone.0183287.s003.doc]

**S1 Table.** PCR conditions for *TERT* and *TERC* amplifications

| **Gene** | **Exon** | **PCR Kit** | **PCR Conditions** |
| --- | --- | --- | --- |
| ***TERT*** | 1 | P.T.* | 94°C 3min + 36x (94°C 45s; 62°C 50s; 72°C 1,5min) |
|  | 2 | P.T.* | 94°C 3min + 35x (94°C 30s; 56°C 50s; 68°C 1min) |
|  | 3 | H.S.** | 94°C 15min + 35x (94°C 30s; 66°C 50s; 68°C 30s) |
|  | 4-6 | P.T.* | 95°C 2min + 35x (95°C 45s; 56°C 50s; 72°C 2min) |
|  | 7-8 | P.T.* | 95° 2min + 35x (95°C 45s; 56°C 30s; 72°C 2min) |
|  | 9 | H.S.** | 95°C 15min + 35x (95°C 30s; 60°C 50s; 72° 1min) |
|  | 10-11 | P.T.* | 95°C 2min + 35x (95°C 30s; 56°C 50s; 72°C 3min) |
|  | 12 | H.S.** | 95°C 15min + 35x (95°C 30s; 64°C 50s; 68°C 1min) |
|  | 13 | H.S.** | 95°C 15min + 35x (95°C 30s; 60°C 50s; 72°C 1min) |
|  | 14-16 | P.T.* | 95° 2min + 35x (95°C 30s; 60°C 50s; 72°C 2min) |
| ***TERC*** | 4 | H.S.** | 95°C 15min + 35x (95°C 30s; 62°C 50s; 72°C 1min) |

P.T.*= Platinum Taq DNA Polymerase (Invitrogen) + PCRx Enhancer System kit (Invitrogen) H.S.**= Hot Start Taq DNA Polymerase (Invitrogen)
