# Supplementary material for: Telomere biology and telomerase mutations in cirrhotic patients with hepatocellular carcinoma
Source: PLoS One. 2017 Aug 16;12(8):e0183287. doi: 10.1371/journal.pone.0183287 (PMC5558955; doi:10.1371/journal.pone.0183287)
Supplement: S2 Table — (DOC) [file pone.0183287.s004.doc]

**S2 Table.** Synonymous and intronic polymorphisms in *TERT* gene

| Exon | Polymorphism | RefSeq ID | Hepatocellular carcinoma samples | | |
| --- | --- | --- | --- | --- | --- |
|  |  |  | Allele frequency (*n*=248) | Number of homozygotes | |
| 2 | Codon 305 GCA/GCG (Ala-Ala) | rs2736098 | 0.129 | 1 | |
|  | IVSE2+39 G>C | rs79662648 | 0.052 | | 0 |
| 3 | IVSE3+130 C>T | rs7725218 | 0.391 | | 18 |
|  | IVSE3+23 C>T | rs191838500 | 0.004 | | 0 |
|  | IVSE3+137 G>A | rs34301490 | 0.020 | | 0 |
| 4 | IVSE3-24 C>T | rs13167280 | 0.153 | | 3 |
|  | IVSE4+10 C>T | rs33948291 | 0.004 | | 0 |
| 7 | IVSE6-10 C>A | N/A | 0.016 | | 0 |
| 14 | Codon 1013 CAC/CAT (His-His) | rs33954691 | 0.109 | | 1 |
| 15 | IVSE14-94 C>T | rs188517214 | 0.004 | | 0 |
|  | IVSE15+32 A>G | rs34742644 | 0.020 | | 0 |
|  | IVSE15+148 A>G | rs369281603 | 0.004 | | 0 |
